# Biological Computation Indexes of Brain Oscillations in Unattended Facial Expression Processing Based on Event-Related Synchronization/Desynchronization

**DOI:** 10.1155/2016/8958750

**Published:** 2016-07-04

**Authors:** Bo Yu, Lin Ma, Haifeng Li, Lun Zhao, Hongjian Bo, Xunda Wang

**Affiliations:** ^1^School of Computer Science and Technology, Harbin Institute of Technology, Harbin 150001, China; ^2^Software College, Harbin University of Science and Technology, Harbin 150001, China; ^3^Brain Research Institute, Beijing Yiran Sunny Technology Co. Ltd., Beijing 100088, China

## Abstract

Estimation of human emotions from Electroencephalogram (EEG) signals plays a vital role in affective Brain Computer Interface (BCI). The present study investigated the different event-related synchronization (ERS) and event-related desynchronization (ERD) of typical brain oscillations in processing Facial Expressions under nonattentional condition. The results show that the lower-frequency bands are mainly used to update Facial Expressions and distinguish the deviant stimuli from the standard ones, whereas the higher-frequency bands are relevant to automatically processing different Facial Expressions. Accordingly, we set up the relations between each brain oscillation and processing unattended Facial Expressions by the measures of ERD and ERS. This research first reveals the contributions of each frequency band for comprehension of Facial Expressions in preattentive stage. It also evidences that participants have emotional experience under nonattentional condition. Therefore, the user's emotional state under nonattentional condition can be recognized in real time by the ERD/ERS computation indexes of different frequency bands of brain oscillations, which can be used in affective BCI to provide the user with more natural and friendly ways.

## 1. Introduction

Recognizing an emotional state of the human user is essential for friendly and natural human-computer interactions. Research in affective Brain Computer Interface (BCI) has significantly increased during the last few years. Moreover, there is growing interest in estimating the user's emotional state from Electroencephalogram (EEG) signals [1–8]. Nevertheless, no previous researches have widely examined the relationship between a user's emotion state of processing Facial Expressions under nonattentional condition and the activities of different brain frequency band.

An important aspect of emotional competence is the ability to recognize emotions from Facial Expressions. It is widely accepted that Facial Expressions play a critical role in social interactions and the ability to quickly and accurately decode Facial Expressions is essential for an individual's successful functioning in a social environment. Converging evidence of affective brain signal revealed that Facial Expressions can be processed under nonattentional condition. Processing unattended Facial Expression usually has some or all of three characteristics, rapidity, unawareness, and automaticity.* (1) Rapidity*. Psychological studies using scalp ERP (Event-Related Potential) [9–14] have suggested that emotive information from Facial Expression was rapidly registered, coded, and categorized, from as early as 100 ms after the appearance of the stimuli.* (2) Unawareness*. Smith [15] observed that subjects' behavioral responses did not differentiate between the affective stimuli in the unaware condition, and activities over the frontal and occipitotemporal brain regions indicated an emotional modulation of the neuronal response by backward masking paradigms. The results reflected that affective faces were processed without conscious awareness at an early stage. Moreover, a recent research suggests that Facial Expressions can be processed rapidly without awareness potentially via a subcortical pathway to the amygdala [16].* (3) Automaticity*. The MMN (Mismatch Negativity) reflects the automatic Facial Expression processing mechanism of the brain. For example, Zhao and Li first observed Facial Expression Mismatch Negativity (EMMN) reflecting the preattentive change detection of Facial Expressions using a modified cross-modal delayed response paradigm, with larger amplitudes for sad than happy expression [17]. Their results firstly evidenced that sad expressions can be automatically processed much more than happy expressions in the absence of focused attention to the face. Chang et al. also found the EMMN for preattentively processing Facial Expression similar to Zhao and Li's finding [18]. Furthermore, Li et al. [19] provided new evidence that Facial Expressions could elicit the preattention memory-comparison-based EMMN. In addition, review [20] concluded that amygdala activation to fearful Facial Expressions appeared completely mandatory under low attentional load conditions, but perhaps not under high attentional load conditions.

It should be noteworthy that most of the previous studies on processing Facial Expressions employed ERP techniques focusing on the detection of phase-locked activities of large neuronal population. Indeed, the ERP analysis did not obtain different brain oscillations and cannot deal with robust event-related dynamical changes possibly relevant to expressional processes. Recently, there was evidence that the EEG oscillation such as theta, alpha, and beta activity is also effective method to study affective processes. For example, Basar et al. [21] demonstrated highly increased occipital theta responses upon stimulation with angry faces. Güntekin and Basar [22] observed that, compared to the happy faces, angry faces evoked higher amplitudes of alpha and beta spectrum. Moreover, Balconi and Pozzoli found that the gamma band activity was varied related to the degree of arousing feature (high or low) of Facial Expressions, whereas the theta band activity was relevant to the emotional significance of faces [23]. To sum up, event-related brain oscillatory responses in theta, alpha, beta, and gamma frequency ranges may be involved in processing facial expression.

In the event-related oscillation analysis, the event-related desynchronization (ERD) reflects the decrease of spectrum power in specific frequency bands and the event-related synchronization (ERS) reflects the increase of spectrum power [24–26]. Several studies analyzed the brain affective oscillation towards processing Facial Expressions and found stronger synchronization (ERS) in delta, theta, and beta band frequencies.* (1) Delta*. Balconi and Lucchiari [27] observed maximal delta synchronization in the posterior regions when participants actively viewed Facial Expression stimuli. They proposed that delta reflected updating of the stimulus. Klados et al. [28] analyzed delta activity evoked by the passive viewing of IAPS pictures and reported delta oscillations were synchronized after emotional stimuli.* (2) Theta*. Aftanas and his colleagues [29, 30] found that the valence discrimination of affective stimuli is associated with the early time-locked synchronized theta activity and emotional stimuli of high and medium arousal also induced greater theta ERS in left anterior and right posterior site. Knyazev et al. [31] showed that in explicit and implicit experiments theta synchronization is stronger upon presentation of emotional stimuli. Similarly, other studies also showed enhanced synchronous activity of theta band for looking at emotional versus neutral faces [27, 31–33].* (3) Alpha*. Sarlo et al. [34] reported that modulation of alpha band ERD associated with watching emotional movie clips. Mu et al. [33] also found that, relative to neutral stimuli, painful stimuli induced alpha-ERD at 200~400 ms in empathy for pain. Moreover, alpha-ERD was modulated by emotional picture content and by picture size [35]. Nevertheless, the previous studies did not clearly state the contribution of alpha-ERD. Recently, Moore et al. [36] observed that mu rhythms were found to respond to the observation of happy and disgusted faces during both empathy and nonempathy tasks with ERD, and the contribution of the mu power suppression response was believed to indicate simulation of the action of producing an observed facial expression.* (4) Beta*. Only several previous studies have shown beta ERS phenomenon of viewing Facial Expression or face. Güntekin and Başar [37] demonstrated that negative emotions are related to increased beta responses, independent of Stimulus Type.

To our knowledge, in the most of previous event-related oscillation studies about processing Facial Expressions, participants were asked to view affective pictures with attention and therefore, the ERD/ERS of each frequency band in processing Facial Expressions under nonattentional condition is still unclear. The present study was conducted in order to establish the relations between each frequency oscillation and nonattentional expressional processes by computing ERD/ERS indexes. We used the deviant-standard-reverse oddball paradigm [38] in which participants engaged in a visual detection task presented in the center of the visual field and the ERD/ERS was compared by physically identical facial stimuli in the visual periphery in order to reduce the influence of low level physical features differences between the faces [39]. This paradigm successfully evoked expressional MMN (EMMN) relative to the preattentive change detection of Facial Expressions [40]. In particular, the deviant-standard-reverse oddball paradigm can investigate the genuine brain oscillations in association with preattentive processing of Facial Expressions.

Some previous studies have evidenced that the schematic face stimuli may evoke the same ERPs and neural responses comparable to those evoked by photographic face ones. For instance, Krombholz et al. [41] concluded that different emotional expressions of schematic faces can modulate the N170 widely regarded as a face sensitive potential. To minimize the variance associated with human face pictures as stimuli, using schematic faces (sad, happy, and neutral) as standard stimuli, Chang et al. found the EMMN similar to Zhao and Li's finding studied by the photographs of a real woman face [18]. Maratos et al. [42] also demonstrated that simple schematic faces evoke neural responses comparable to those evoked by photographic face stimuli. They argued that schematic visual stimuli may still validly represent face processing. Importantly, significantly increased fMRI signals were found in the amygdala, hippocampus, and prefrontal cortex in response to emotional versus neutral schematic faces, suggesting that schematic faces may be useful for studying brain responses to emotional stimuli because of their simplicity relative to human faces. Therefore, in the present study, to minimize the variance associated with genuine facial photographs as stimuli, we used schematic sad, happy, and neutral faces as deviant and standard stimuli in different blocks under a situation where the faces themselves were unrelated to the participant's task. We expected that ERD/ERS computation indexes in some frequency band would be different for processing happy and sad expressions in preattentive stage.

The aim of our goal is to detect users' emotion state during preattentive processing of emotional faces through the ERD/ERS computation indexes in brain oscillations containing the corresponding Facial Expression processing information. Brain oscillations represent a wealth of cognitive information. Several studies reported that the brain oscillations can process Facial Expressions when the users focus their attention on the affective stimuli. But the function of brain oscillations in unattended Facial Expressions processing has not been studied. The significance of our study is to detect the users' emotion state by brain oscillations when the users nonattentively process the affective stimuli shown on the computer.

## 2. EEG Experiments and Computation Methods

### 2.1. Subjects

We randomly chose fourteen college students (seven females and seven males; mean age = 23.85 years, standard deviation = 2.91) and invited them to participate in the experiment. Thirteen participants were right-handed and only one female participant was left-handed. All subjects completed at least 10 years of education. All participants had normal or corrected to normal visual acuity. They were free of current past neurological or psychiatric disorders. And participants signed their informed consent before the start of the experiment. All researches involving human participants have been approved by applied ethics research center, Harbin Industrial University.

### 2.2. Stimuli

**Figure 1:**
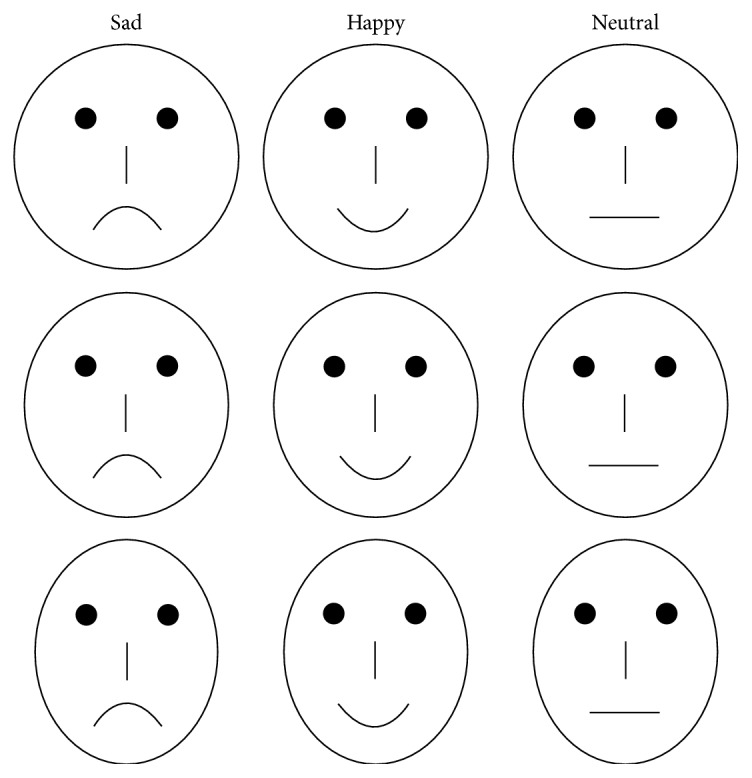
Samples of schematic faces with sad, happy, and neutral expressions.

**Figure 2:**
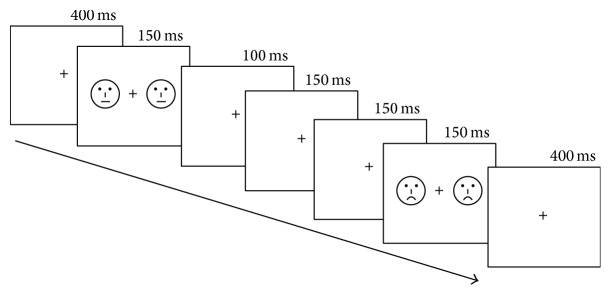
Examples of the sequences.

In all experiments, 54 schematic faces with happy, sad, and neutral expressions were presented (see Figure 1). Each type of expression included 18 schematic faces, which changed randomly in facial feature and the distance of eyes. Two schematic faces with the same expression were presented peripherally with a cross in the center on each screen for 150 ms, with a visual angle of 3.68 × 3.42 from a viewing distance of 70 cm. This screen was followed by an interstimulus interval 400 ms during which the size of a cross has changed. The cross was continuously presented at the center of the screen. Participants were required to focus on the central fixation cross and make a speeded button-press when the cross changed in size (see Figure 2).

### 2.3. Procedure

The deviant-standard oddball and deviant-standard-reverse oddball paradigm were used in the experiment. There were four block types in the current experiment: (a) happy faces as deviant stimuli and neutral faces as standard stimuli; (b) the probability of face of the block (a) which is reversed, happy faces as standard stimuli, and neutral faces as deviant stimuli; (c) sad faces as deviant stimuli and neutral faces as standard stimuli; (d) the probability of face of the block (c) which is reversed, sad faces as standard stimuli, and neutral faces as deviant stimuli. For each blocked condition, there were three sequences with 104 standard faces and 30 deviant faces. Ten standard stimuli (+) were presented in the head of each sequence in order to establish the sensory memory pattern. There were totally 161 trials for each sequence. In each sequence, the selection of face and the change of the cross were pseudorandom to reduce interactions between Facial Expression and learning habituation. The order of block conditions was counterbalanced across participants. Presenting the same faces as both deviant and standard stimuli allowed us to investigate the genuine brain oscillation by comparing physically identical stimuli, thereby reducing the influence of low level physical features differences between the neutral face and the sad (or happy) face. The participants were seated in a reclining chair in a sound-attenuated and electrically shielded room and instructed to focus on the center of the visual field, ignoring the same faces which appeared on both sides of the cross and to detect unpredictable changes in size of the fixation as accurately as possible. They were also asked to minimize any eye movement during each block.

### 2.4. Recordings

EEG was continuously recorded with a band pass filter from 0.05 Hz to 150 Hz at a sampling rate of 500 Hz by Neuroscan Synamps2 Amplifier. An ElectroCap with 64 Ag/AgCl electrodes was used to record EEG from active scalp sites referred to the tip of the nose (international 10/20 system of electrode placement). Vertical Electrooculography (VEOG) and Horizontal Electrooculography (HEOG) were recorded with two pairs of electrodes, one placed above and below right eye and the other 10 mm from the lateral canthi. All electrode impedance was less than 5 kΩ throughout the experiment.

### 2.5. Data Analysis by ERD/ERS

All data were processed by Neuroscan Edit software. EEG automatic procedure was programed by TCL (Tool Command Batch Processing Language). The raw EEG contaminated by amplifier clipping, bursts of electromyographic activity, or peak-to-peak deflection exceeding ±100 *μ*v were excluded from the raw EEG. Firstly, we remove the VEOG from the raw EEG signals. Then the EEG was segmented into the epoch from −50 ms before stimulus to 400 ms after stimulus. Successively, the baseline of the epoch EEG was corrected. After EOG correction and baseline correction, we reject the trial in which the voltage exceeds the defined criteria (70~150 *μ*v). The rejection rate is less than 20 percent. Only artifact-free trials were considered in the following steps.

We used the classical method to compute the time course of ERD proposed by Pfurtscheller and da Silva [26]. Firstly, the digital EEG data were band pass filtered in the following frequency bands: delta (1–4 Hz), theta (4–8 Hz), alpha 1 (8–10 Hz), alpha 2 (10–13 Hz), beta 1 (13–20 Hz), and beta 2 (20–30 Hz) (48 dB/oct). Complex demodulation was used to obtain the given bandwidth [43]. Secondly, the filtered signal samples were squared to obtain power samples. Thirdly, an average absolute power value for each electrode under different conditions (sad faces as standard stimuli, sad faces as deviant stimuli, happy faces as standard stimuli, and happy faces as deviant stimuli) was calculated separately for each frequency band within subject. We obtained a time course of band power values, including phase-locked (evoked) and not phase-locked power (induced) changes as well. At last, −50~0 ms before stimulus was used as the reference interval to obtain percentage values for ERD/ERS. Changes in a band power were defined as the percentage of a decrease (ERD) in the band power during a test interval (here 0~400 ms after stimulus) compared to a reference interval, according to the following equation:

(1) $$\begin{array}{c} ERD\%=\frac{\left[\left(\text{band power of reference interval}\right)-\left(\text{band power of test interval}\right)\right]}{\left(\text{band power of reference interval}\right)*100\%}. \end{array}$$

Positive ERD indicates a power decrease and negative ERD a power increase (ERS). For each subject, the ERD waveform of each frequency band was divided into different time intervals according to the shape and peaks of the ERD waveform. The average ERD values across the respective Electrode Sites were calculated for each time interval.

**Figure 3:**
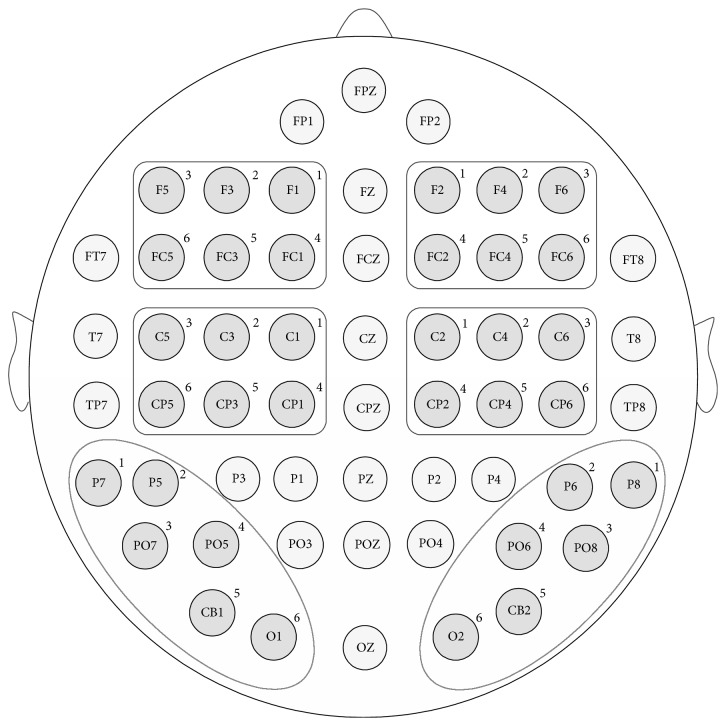
Electrode Sites in different Brain Areas.

The software SPSS was used for statistical analysis. The statistical analysis was based on within subject factorial models. The average ERD values across the respective Electrode Sites for each time interval were entered into repeated measures analysis of variance (ANOVA) with five repeated factors: Facial Expression (happy, sad) × Stimulus Type (standard, deviant) × Brain Area (frontal area, central area, and parietal-occipital area) × Hemisphere (left, right) × Electrode Sites (1, 2, 3, 4, 5, and 6, see Figure 3). Six individual Brain Areas (left frontal area, right frontal area, left central area, right central area, left parietal-occipital area, and right parietal-occipital area) are analyzed using three repeated factors: Facial Expression (happy, sad) × Stimulus Type (standard, deviant) × Electrode Sites (1, 2, 3, 4, 5, and 6, see Figure 3). We use Mauchly's test to test the hypothesis that the variances of the differences between conditions are equal (sphericity). If sphericity assumption is violated, Huynh-Feldt correction should be used to correct degrees of freedom when the estimates of sphericity are greater than 0.75, and the Greenhouse-Geisser correction should be used instead when sphericity estimates are less than 0.75. If one factor with more than two levels has the main effect, post hoc analysis was conducted using the Bonferroni test. If the interactions between factors exist, simple effect analysis was conducted to look at the effect of one factor at individual levels of the other factors.

## 3. ERD/ERS Computation Indexes Results

The accuracy of target stimuli was above 90% for all conditions, indicating the degree of attention at high level.  Table 1 shows the statistical analysis results in each frequency band. The detailed ERD/ERS computation indexes results of each frequency are as follows.

### 3.1. ERD/ERS in Delta Band (1–4 Hz)

**Figure 4:**
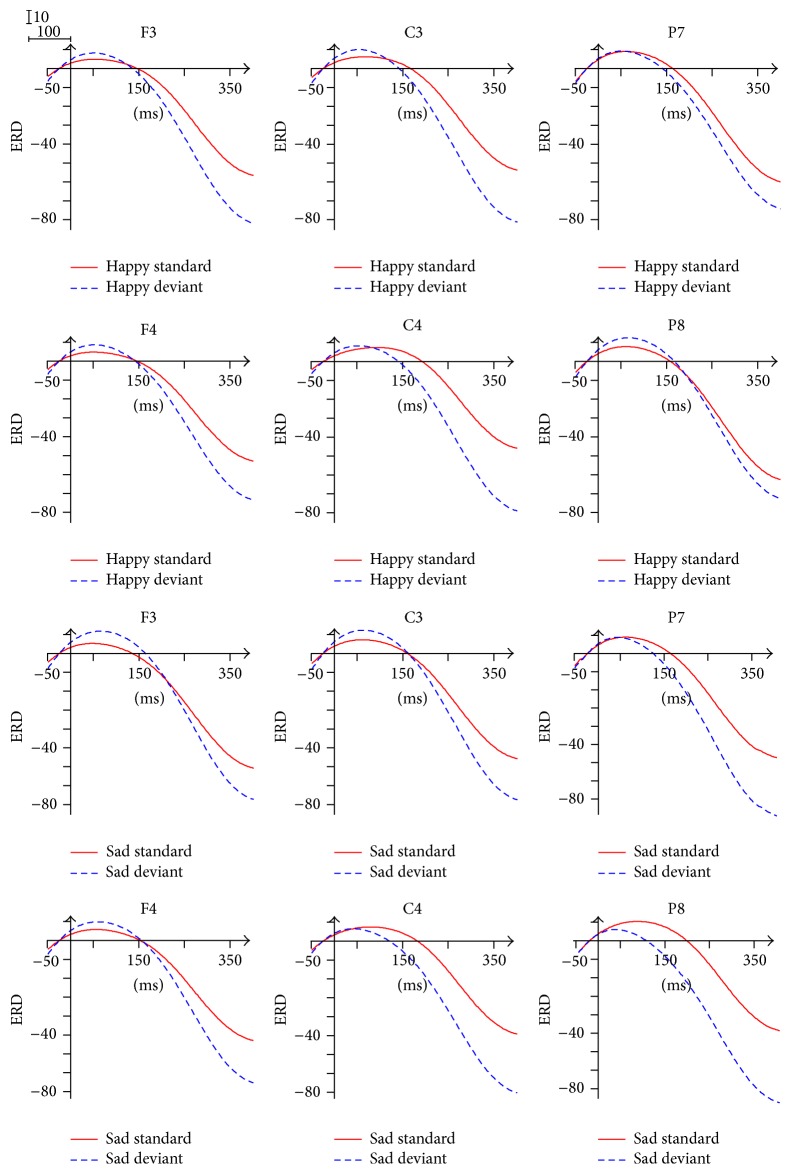
ERD/ERS in delta frequency band.

The ERD/ERS waveform of delta band was divided into four time windows: 0~100 ms, 100~200 ms, 200~300 ms, and 300~400 ms. As shown in Figure 4, ERD phenomenon was found in the 0~150 ms, while ERS was found in the following time interval 150~400 ms.

For the analysis of ERD/ERS in the time of 0~100 ms after stimulus onset, the ANOVA analysis revealed a significant interaction of Stimulus Type × Brain Area (*F*(1.396,18.147) = 4.449, *p* = 0.038), indicating larger ERD of deviant stimuli (8.445) compared with the ERD of standard stimuli (5.118) in the frontal area. The successive analysis indicated that the ERD difference between Brain Areas is significant only for the standard stimuli, with the largest ERD in the parietal-occipital area (8.707). In addition, the interaction of Facial Expression × Brain Area (*F*(2,26) = 5.082, *p* = 0.014) was significant within the 0~100 ms, reflecting the significant Brain Area difference of ERD only in response to happy expressions (*p* = 0.004), with the biggest ERD (9.109) in the parietal-occipital area. Individual Brain Area analysis showed the main effect of Stimulus Type in the left frontal area (*F*(1,13) = 12.061, *p* = 0.004), with significantly greater ERD of deviant stimuli (8.987) relative to the ERD of standard stimuli (5.001). In the 100~200 ms, the ANOVA analysis for the individual Brain Area also showed the main effect of Stimulus Type in the right central area (*p* = 0.048) and the left parietal-occipital area (*p* = 0.039), with ERD for standard stimuli and ERS for deviant stimuli.

The main effect of Stimulus Type was found within the 200~300 ms (*F*(1,13) = 11.710, *p* = 0.005) and the 300~400 ms (*F*(1,13) = 14.449, *p* = 0.002), with greater ERS of deviant stimuli relative to the ERS of standard stimuli. In the 200~300 ms and the 300~400 ms, the ANOVA analysis for the individual Brain Area also showed the main effect of Stimulus Type in the six individual Brain Areas, with greater ERS of deviant stimuli in comparison with the ERS of standard stimuli. In addition, the ANOVA analysis revealed a significant interaction of Facial Expression × Stimulus Type × Brain Area within the 200~300 ms (*F*(2,26) = 4.366, *p* = 0.023) and the 300~400 ms (*F*(2,26) = 3.811, *p* = 0.035), indicating that, in response to sad expressions, the ERS of deviant stimuli was significantly larger than the ERS of standard stimuli in the frontal area, the central area, and the parietal-occipital area. The further analysis within the 300~400 ms indicated that, in response to happy expressions, the ERS of deviant stimuli was significantly larger than the ERS of standard stimuli in the frontal area (*p* = 0.031) and the central area (*p* = 0.045).

### 3.2. ERD/ERS in Theta Band (4–8 Hz)

**Figure 5:**
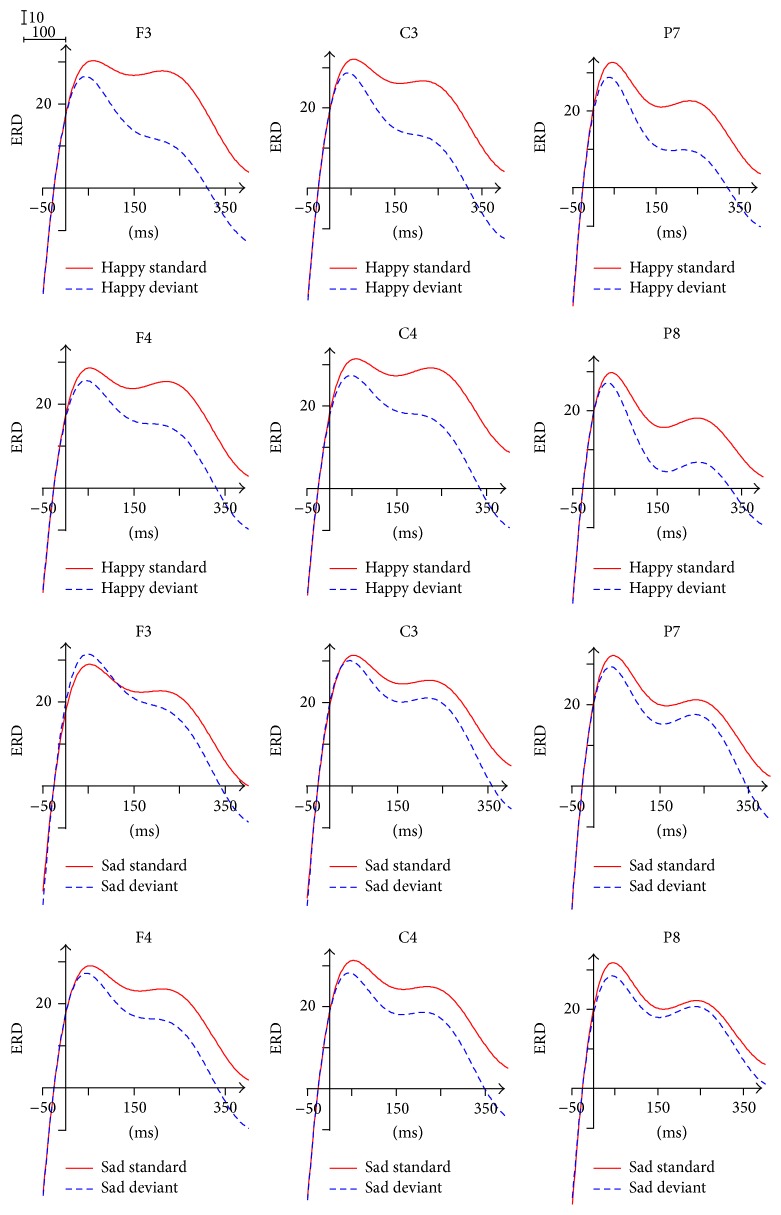
ERD/ERS in theta frequency band.

The ERD/ERS waveform of theta band was divided into four time windows: 0~100 ms, 100~200 ms, 200~300 ms, and 300~400 ms. As shown in Figure 5, we observed significant desynchronization and two peaks (the first within the 40~60 ms and the second within the 210~250 ms) in the waveform.

The main effect of Stimulus Type was found within the 0~100 ms (*F*(1,13) = 5.429, *p* = 0.037), the 100~200 ms (*F*(1,13) = 9.201, *p* = 0.010), and the 200~300 ms (*F*(1,13) = 8.929, *p* = 0.010), with significantly smaller ERD of deviant stimuli in comparison with the ERD of standard stimuli. The effect of Stimulus Type was also significant within the 300~400 ms (*F*(1,13) = 10.009, *p* = 0.007), with smaller ERS of deviant stimuli in comparison with the ERD of standard stimuli. In addition, the ANOVA analysis for the individual Brain Area showed the main effect of Stimulus Type (*p* < 0.05) in the left (right) frontal area (100~200 ms, 200~300 ms, and 300~400 ms), the left central area (100~200 ms, 200~300 ms, and 300~400 ms), the right central area (0~100 ms, 100~200 ms, 200~300 ms, and 300~400 ms), and the left parietal-occipital area (200~300 ms, 300~400 ms), with significantly smaller ERD of deviant stimuli than the ERD of standard stimuli.

### 3.3. ERD/ERS in Alpha 1 Band (8–10 Hz)

**Figure 6:**
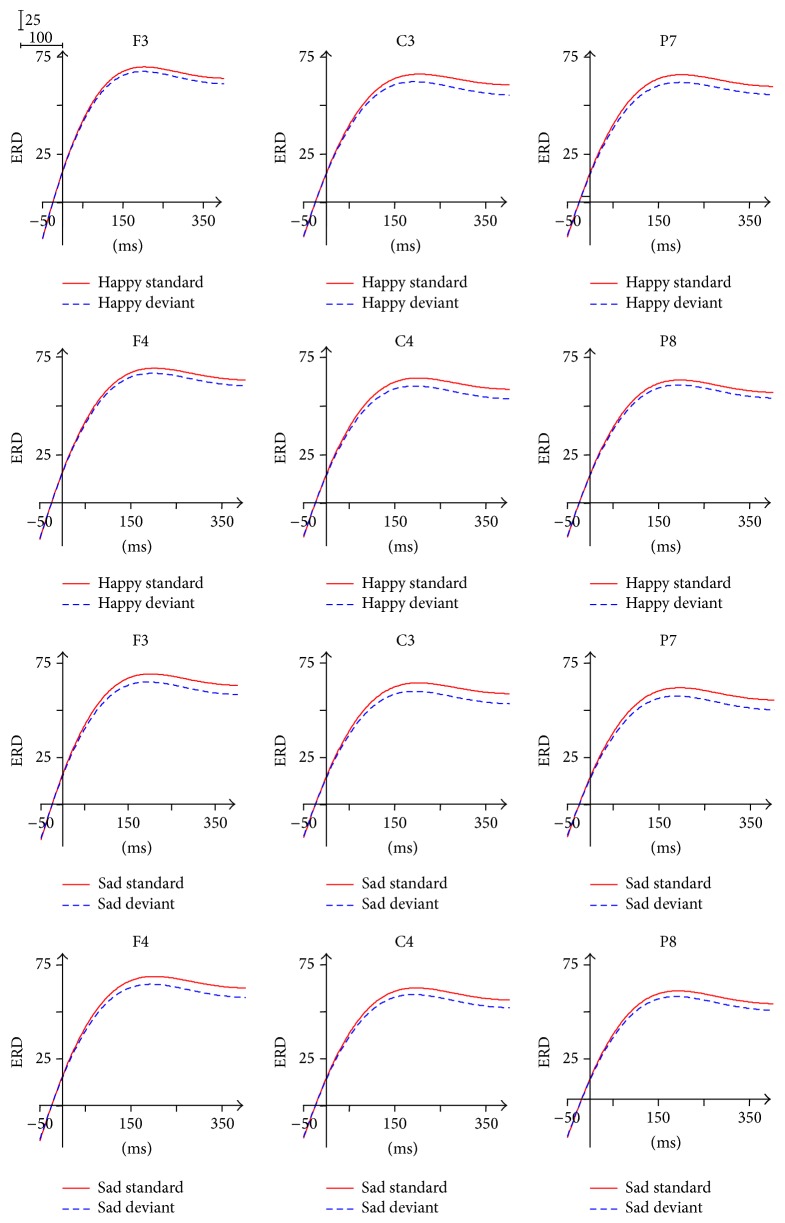
ERD/ERS in alpha 1 frequency band.

The ERD/ERS waveform of alpha 1 band was divided into three time windows: 0~100 ms, 100~250 ms, and 250~400 ms. As shown in Figure 6, we observed significant desynchronization and only one peak at about 200 ms.

The ANOVA analysis applied to each time window showed the statistical significance of Brain Area effect (*p* < 0.05), with the biggest ERD in the frontal area. Post hoc tests revealed that the effect was due to the significant ERD differences between the frontal area and the central area within the 0~100 ms and between the frontal area and the parietal-occipital area within the 100~250 ms and the 250~400 ms.

### 3.4. ERD/ERS in Alpha 2 Band (10–13 Hz)

**Figure 7:**
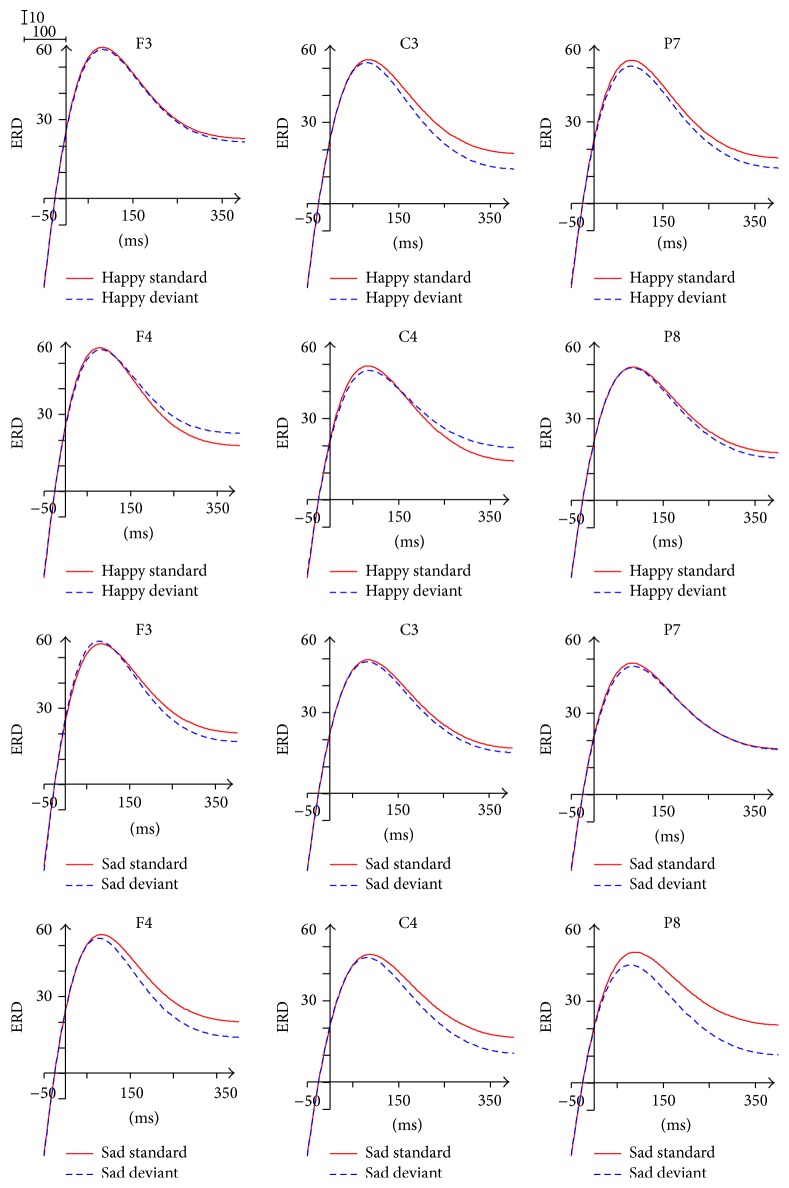
ERD/ERS in alpha 2 frequency band.

The ERD/ERS waveform of alpha 2 band was divided into three time windows consistent with alpha 1. As shown in Figure 7, we observed significant desynchronization and only one peak at about 82 ms.

For the analysis of ERD/ERS in the time of 0~100 ms after stimulus onset, the main effect of Facial Expression (*F*(1,13) = 5.618, *p* = 0.034) was found, reflecting higher ERD in response to happy expressions (43.938) in comparison with the ERD in response to sad expressions (41.675). In addition, the ANOVA analysis for the individual Brain Area showed the main effect of Facial Expression in the left central area (*p* = 0.010) (0~100 ms), also reflecting that the ERD in response to happy expressions (43.785) was significantly larger than the ERD in response to sad expressions (40.518). The significant effect of Hemisphere was also found (*F*(1,13) = 16.334, *p* = 0.001) within the 0~100 ms, with higher ERD of the left Hemisphere (43.609) in comparison with the ERD of the right Hemisphere (42.004).

There was a significant effect of Brain Area within the 0~100 ms (*F*(2,26) = 20.467, *p* = 0.000) and the 100~250 ms (*F*(2,26) = 6.552, *p* = 0.005), with the biggest ERD in the frontal area and the smallest ERD in the parietal-occipital area. Post hoc tests revealed the significant ERD differences between the frontal area and the central area and between the frontal area and the parietal-occipital area.

The interaction of Facial Expression × Stimulus Type within the 100~250 ms (*p* = 0.045) in the right parietal-occipital area and Brain Area × Hemisphere (*p* = 0.043) within the 250~400 ms was found, but the simple effect analysis applied to these interactions showed no significance.

### 3.5. ERD/ERS in Beta 1 Band (13–20 Hz)

**Figure 8:**
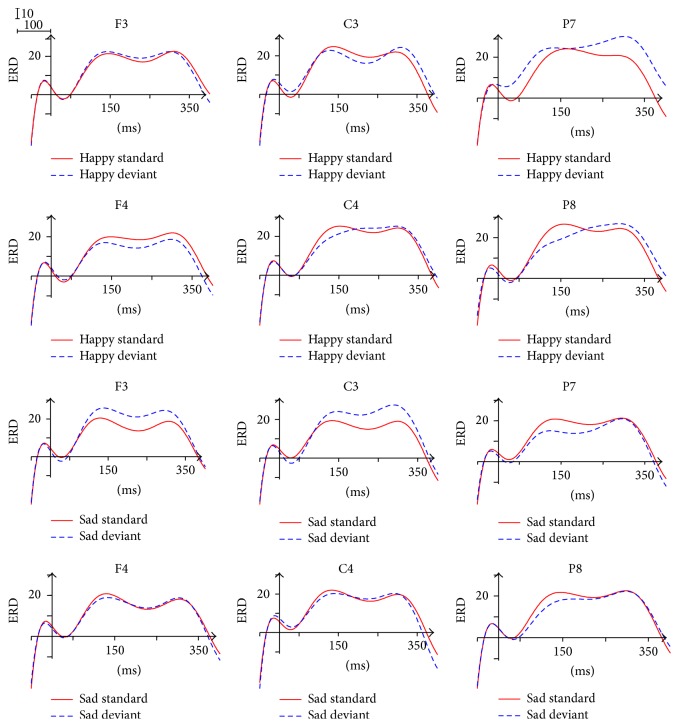
ERD/ERS in beta 1 frequency band.

The ERD/ERS waveform of beta 1 band was divided into five time windows: 50~150 ms, 150~250 ms, 250~300 ms, 300~350 ms, and 350~400 ms. As shown in Figure 8, we observed significant desynchronization and two peaks (the first within the 120~150 ms and the second within the 290~320 ms) in the waveform.

The main effect of Facial Expression was found within the 350~400 ms (*F*(1,13) = 5.089, *p* = 0.042), with higher ERD in response to happy expressions (6.270) in comparison with the ERD in response to sad expressions (0.413). The ANOVA analysis for the individual Brain Area showed the significant effect of Facial Expression (*p* < 0.05) in the right central area (150~250 ms), the left parietal-occipital area (150~250 ms, 300~350 ms), and the right parietal-occipital area (150~250 ms), also reflecting higher ERD in response to happy expressions than the ERD in response to sad expressions.

The significant interaction of Facial Expression × Stimulus Type × Brain Area was found within the 250~300 ms (*F*(1.350,17.551) = 4.039, *p* = 0.050), the 300~350 ms (*F*(1.327,17.251) = 4.510, *p* = 0.039), and the 350~400 ms (*F*(1.339,17.410) = 4.776, *p* = 0.034). The simple effect analysis indicated that, only in response to happy expressions, the ERD of deviant stimuli was significantly larger than the ERD of standard stimuli in the parietal-occipital area (*p* < 0.05).

The 150~250 ms time window showed a significant interaction of Facial Expression × Brain Area (*F*(2,26) = 3.608, *p* = 0.041), indicating that the ERD in response to happy expressions (24.813) was significantly larger than the ERD in response to sad expressions (18.774) in the parietal-occipital area (*p* = 0.01).

The ANOVA analysis did not reveal any significant effect for the 50~150 ms time interval.

### 3.6. ERD/ERS in Beta 2 Band (20–30 Hz)

**Figure 9:**
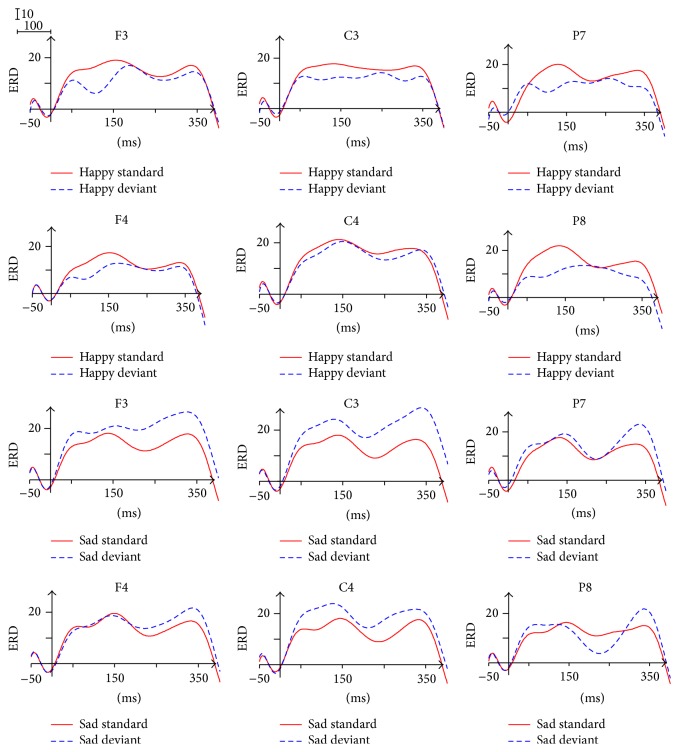
ERD/ERS in beta 2 frequency band.

The ERD/ERS waveform of beta 2 band was divided into five time windows consistent with beta 1. As shown in Figure 9, we also observed significant desynchronization and two peaks (the first within the 130~150 ms and the second within 320~350 ms) in the waveform.

The ANOVA analysis for the individual Brain Area showed the main effect of Facial Expression in the left frontal area (50~150 ms, 300~350 ms) and the left central area (300~350 ms), reflecting that the ERD in response to sad expressions was significantly larger than the ERD in response to happy expressions. The ANOVA analysis did not reveal any significant effect for the 150~250 ms time window.

A significant interaction of Facial Expression × Stimulus Type × Brain Area (*F*(2,26) = 3.644, *p* = 0.040) was found within the 250~300 ms. The simple effect analysis indicated that, only in response to sad expressions, the ERD of deviant stimuli was significantly larger than the ERD of standard stimuli in the central area (*p* < 0.05). In addition, the interaction of Facial Expression × Stimulus Type (*p* < 0.05) was significant in the left frontal area (250~300 ms), the left central area (250~300 ms, 300~350 ms, and 350~400 ms), and the whole Brain Area (300~350 ms). The simple effect analysis also indicated that, only in response to sad expressions, the ERD of deviant stimuli was significantly larger than the ERD of standard stimuli (*p* < 0.05).

## 4. Discussion

Our goal in this paper is to find biological computation indexes of brain oscillations for detecting the user's emotional state under nonattentional condition, which can be used in affective BCI to provide the user with more natural and friendly ways. We investigated the different ERD/ERS computation indexes of typical frequency bands for processing Facial Expressions under nonattentional conditions using a deviant-standard-reverse oddball paradigm. As we expected, ERD/ERS computation indexes were modulated by experimental conditions. ERS was only observed in delta band, with greater ERS of deviant stimuli relative to the ERS of standard stimuli. But for theta band, deviant stimuli elicited smaller ERD than standard stimuli. Facial Expressions also modulated ERD. Higher ERD in response to happy expressions in comparison with the ERD in response to sad expressions was found in alpha 2 band. Two important results were found in beta 1. Firstly, in response to happy expression, the ERD of deviant stimuli was significantly larger than the ERD of standard stimuli. Secondly, the ERD in response to happy expressions was significantly larger than the ERD in response to sad expressions. Excitingly, corresponding results were obtained in beta 2. Firstly, only in response to sad expression, the ERD of deviant stimuli was significantly larger than the ERD of standard stimuli. Secondly, the ERD in response to sad expressions was significantly larger than the ERD in response to happy expressions.

In the present study, we used the deviant-standard-reverse oddball paradigm in which the probability of both stimuli is reversed [38]. Using this paradigm, Jacobsen and Schröger obtained the genuine duration MMN and proved that subjects detected deviations in sound duration without necessarily becoming aware of it. And the participants were instructed to focus on the center of the visual field, ignoring the same faces which appeared on both sides of the cross, and to detect unpredictable changes in size of the fixation as accurately as possible. Using the similar experiment design, Stefanics et al. [39] studied the processing of unattended facial emotions by visual MMN. To minimize the variance information associated with a real human face, we used schematic sad and happy faces as the stimulus different from the stimulus in the experiments of Stefanics. Therefore, the selection of the paradigm and the design of the experiment process ensured the subjects to process the Facial Expressions unconsciously in nonattentional condition.

Many studies have shown that brain oscillations are associated with the processing of Facial Expressions. However, little is known about the dynamic properties of brain oscillations in nonattentional condition. Although expressional MMN could indicate the dynamic processing of facial processing in nonattentional condition, these measures confuse several different frequency bands, not reflecting the real brain oscillations. It has been evidenced that ERS and ERD of each frequency band could reflect relative small differences in the processing of Facial Expressions [27, 33, 37, 44]. Thus, the present study generalizes this finding to processing Facial Expressions in nonattentional condition, using a deviant-standard-reverse oddball paradigm. The analysis results demonstrated that ERD/ERS computation indexes of each frequency band in automatically and preattentively processing Facial Expressions indeed existed, and ERD/ERS computation indexes of in some frequency bands were different for happy and sad expressions. Therefore, the user's emotional state under nonattentional condition can be recognized by the ERD/ERS computation indexes of different frequency bands, which can be used in affective BCI to provide the user with more natural and friendly ways.

### 4.1. Delta Activity (1–4 Hz)

ERS within 150~400 ms was found in processing Facial Expressions under nonattentional condition, in concert with previous evidences that synchronized delta power after the onset of actively viewing Facial Expression stimuli [27] or passive viewing emotionally evocative IAPS pictures [28]. The amplitude of the delta response is considerably increased during oddball paradigms [24, 27]. Moreover, Balconi and Pozzoli found that delta varied as a function of the necessity of stimulus evaluation and memory updating [23]. Delta synchronization of the results reflected updating of the Facial Expressions under nonattentional condition. Therefore, delta activity can monitor the salience of the Facial Expressions (sad and happy). Unlike the previous studies, ERD phenomenon was found in the 0~150 ms, due to the recognition of different Stimulus Type (standard and deviant) in the reversed oddball paradigm. The greater ERD/ERS of deviant stimuli in comparison with the standard ones suggested that delta is more relevant to the processing of deviant stimulus. In addition, in response to both happy expressions and sad expressions, the ERS of deviant stimuli was significantly larger than the ERS of standard stimuli. It indicated that delta was involved in the initial processing of both Facial Expressions, but not distinguishing the difference between happy and sad expressions.

### 4.2. Theta Activity (4–8 Hz)

ERD was found in this band instead of ERS that had been reported in literatures [27, 29, 31, 32, 44]. The factors of emotional stimulus, the attending degree of consciousness, experimental process, and analysis method may lead to different experiment results. Klimesch concluded that the encoding of new information is reflected by theta oscillations in hippocampo-cortical feedback loops [45]. The ERD of theta might correlate with active inhibition to process peripheral Facial Expressions automatically and preventing attention from being unnecessarily allocated to those stimuli. The inhibition for the standard is greater than the deviant. In other words, theta pays more attentional resource to the deviant relative to the standard. Therefore, using the ERD analysis, we conclude that theta plays a role of discriminating Stimulus Type of facial expression.

### 4.3. Alpha Activity (8–13 Hz)

The ERD found in alpha 1 (8–10 Hz) and alpha 2 (8–13 Hz) band during processing Facial Expressions under nonattentional condition was consistent with the previous findings that the ERD of alpha was found during viewing emotional stimuli [32, 34–36]. For alpha 1 (0~400 ms) and alpha 2 (0~250 ms) band, we found the biggest ERD in the frontal area in comparison with the central area and the parietal-occipital area. Contrary to the ERD of theta, the ERD of alpha is usually interpreted as a correlate of an activated cortical area, reflecting gradual release of inhibition associated with the emergence of complex cognitive processes [46]. The function of desynchronization in low alpha band is associated with attentional process [26, 46–48], whereas desynchronization in high alpha band is related to memory and semantic processing demands [45, 49–51]. In the processing of unattended Facial Expressions, alpha 1 band plays a part in the maintenance of attention to the stimuli, the cross at the center of the screen, and neglecting the surrounding Facial Expressions. Alpha 2 band is mainly associated with long-term memory. The ERD of alpha 2 reflects the retrieval of the semantic information about emotional memory to process the peripheral Facial Expressions. Therefore, processing unattended Facial Expressions is a preattentive and automatic mechanism operating on a semantic information level, and the ERD of alpha 2 reflects the activation of semantic memory system. The results also suggested that the desynchronization of alpha 1 and alpha 2 in the frontal area was attributed to more cognitive involvement during the processing of unattended Facial Expressions than the central area and the parietal-occipital area. In the time window 0~100 ms of alpha 2, the ERD in response to happy expressions was higher than the ERD in response to sad expressions in the left central area, which is consistent with the well-known conclusion that the left brain Hemisphere is more involved in positive emotion and the right Hemisphere more in negative emotion [52]. However, unexpectedly, the significant main effect of Hemisphere was found, with higher ERD of the left Hemisphere in comparison with the ERD of the right hemisphere. It was evidenced that, by ERD analysis, the left and right lobes participated in valence discrimination of emotional stimulus [53]. A new study has drawn the conclusion that the left brain may be “emotional” [54]. Therefore, we tentatively pointed out that the left brain also attended the processing of unattended Facial Expressions.

### 4.4. Beta Activity (13–30 Hz)

The beta band is usually desynchronized during motor tasks and synchronized shortly after movement [55]. Therefore, the perception of Facial Expression processing based on a sensorimotor mirroring mechanism may lead to the ERD phenomenon of beta instead of the ERS phenomenon of beta found in the previous studies. The most important finding in this band is that in beta 1, in response to happy expressions, the ERD of deviant stimuli was significantly larger than the ERD of standard stimuli in the parietal-occipital (250~300 ms, 300~350 ms, and 350~400 ms), whereas in beta 2, in response to sad expressions, the ERD of deviant stimuli was significantly larger than the ERD of standard stimuli in the frontal area and the central area. In the deviant-standard-reverse oddball paradigm, the difference between the deviant stimuli and the standard ones of this band represented the genuine oscillation responses in the processing of unattended Facial Expressions. The ERD computation indexes between beta 1 and beta 2 could be tentatively interpreted as brain responsiveness related to Facial Expression differentiation processes (happy versus sad). Accordingly, we can conclude that beta 1 band is more pertinent to the mechanism of happy processing whereas beta 2 band is more relevant to the mechanism of sad processing. Beta band can recognize the Facial Expression through comparing the retrieval of semantic information about emotional memory caused by alpha band.

Before concluding, we would like to reiterate three procedural decisions that constrained the interpretation of the present findings. Due to the fact that emotional information of the schematic faces is obvious, we used schematic faces as facial stimuli in order to eliminate the influence of irrelevant information from faces (e.g., age, gender). Although many previous studies demonstrated the availability of schematic faces, it is necessary to further investigate the hypothesis using the genuine faces in order to get more general evidence. Furthermore, gender maybe acts as a factor in Facial Expression processing. Previous study indicated that occipital beta response for female was significantly larger than for male in the Facial Expression processing under aware conditions [56]. Gender differences in unaware condition could be considered in the later study. In addition, only one type of negative Facial Expression (sad) was used in this study. There was evidence that processing negative expressions are modulated by emotional arousal. For example, some studies found differences in processing sad faces and other negative Facial Expressions (e.g., anger, fear). So ERD/ERS studies using multiexpressions under nonattentional condition await further investigation.

## 5. Conclusion

Our findings emphasize the importance that ERD/ERS computation indexes could be used to investigate automatic facial emotional processing. Particularly, delta and theta are used to update Facial Expressions and distinguish the standard stimuli and the deviant ones, while alpha 2, beta 1, and beta 2 band are relevant to Facial Expression processing. The ERD of alpha 2 band reflects the retrieval of the semantic information about emotion to process the peripheral Facial Expressions. Beta 1 and beta 2 band can, respectively, recognize happy and sad Facial Expressions through comparing the retrieval of semantic information about emotional memory caused by alpha band. The present study first gave the contribution of each frequency band for comprehension of Facial Expressions in preattentive stage. The ERD/ERS computation indexes of different frequency bands can be used to automatically recognize the emotional state of users from their EEG under nonattention affective BCI. Our next work is single-trial classification for affective BCI using ERD/ERS computation indexes of different frequency band.

## Figures and Tables

**Table 1:** ERD/ERS computation indexes results in each frequency band, containing main effects and interaction effects in each time interval (ms).

Frequency band	Interval (ms)	Main effect	Interaction effect
Delta (1–4 Hz)	0~100	Stimulus Type in the left frontal	Facial Expression × Brain Area Stimulus Type × Brain Area
	100~200	Stimulus Type in the right central and left parietal-occipital	Facial Expression × Brain Area
	200~300	Stimulus Type Stimulus Type in individual Brain Area	Facial Expression × Stimulus Type × Brain Area
	300~400	Stimulus Type Stimulus Type in individual Brain Area	Facial Expression × Stimulus Type × Brain Area

Theta (4–8 Hz)	0~100	Stimulus Type Stimulus Type in the right central	
	100~200	Stimulus Type Stimulus Type in the left (right) frontal and the left (right) central	
	200~300	Stimulus Type Stimulus Type in the left (right) frontal, the left (right) central, and the left parietal-occipital	
	300~400	Stimulus Type Stimulus Type in the left (right) frontal, the left (right) central, and the left parietal-occipital	

Alpha 1 (8–11 Hz)	0~100	Brain Area	
	100~250	Brain Area	
	250~400	Brain Area	

Alpha 2 (11–13 Hz)	0~100	Facial Expression Brain Area Hemisphere Facial Expression in the left central	
	100~250	Brain Area	Facial Expression × Stimulus Type in the right parietal-occipital
	250~400		Brain Area × Hemisphere

Beta 1 (13–20 Hz)	50~150		
	150~250	Facial Expression in the right central and the left (right) parietal-occipital	Facial Expression × Brain Area
	250~300	Stimulus Type in the left central	Facial Expression × Stimulus Type × Brain Area
	300~350	Facial Expression in the left parietal-occipital	Facial Expression × Stimulus Type × Brain Area
	350~400	Facial Expression	Facial Expression × Stimulus Type × Brain Area

Beta 2 (20–30 Hz)	50~150	Facial Expression in the left frontal	
	150~250		
	250~300		Facial Expression × Stimulus Type × Brain Area Expression × Stimulus Type in the left frontal (central)
	300~350	Facial Expression in the left frontal (central)	Facial Expression × Stimulus Type Facial Expression × Stimulus Type in the left central
	350~400	Stimulus Type in the left central	Facial Expression × Stimulus Type in the left central
